# Validation of the minimum dietary diversity for women as a predictor of micronutrient adequacy among lactating women in Ethiopia

**DOI:** 10.3389/fnut.2024.1459041

**Published:** 2024-09-19

**Authors:** Yonatan Menber, Selamawit Gashaw, Tefera Belachew, Netsanet Fentahun

**Affiliations:** ^1^Department of Nutrition and Dietetics, School of Public Health, College of Medicine and Health Sciences, Bahir Dar University, Bahir Dar, Ethiopia; ^2^Department of Nutrition and Dietetics, Faculty of Public Health, College of Public Health, Jimma University, Jimma, Ethiopia

**Keywords:** minimum dietary diversity, nutrient adequacy, validation, lactating women, Ethiopia

## Abstract

**Background:**

The Minimum Dietary Diversity for Women (MDD-W) indicator is used as a proxy indicator for assessing micronutrient adequacy among women of the reproductive age group. Variations were observed in studies, and there was also a lack of evidence regarding the performance of this proxy indicator in Ethiopia, a country with diverse dietary consumption practices. This study aimed to validate the performance of the MDD-W in predicting micronutrient intake adequacy among lactating women in Ethiopia.

**Methods and materials:**

A community-based cross-sectional study was conducted among randomly selected 457 lactating women in Northwest Ethiopia from February 2 to 18, 2023. A multistage sampling technique was used to select 457 study participants. A single multiphasic interactive 24-h dietary recall was used to collect dietary intake data. Ten food groups were used to compute the Minimum Dietary Diversity for Women, and the Mean Adequacy Ratio was used to assess nutrient intake adequacy. Spearman’s rank correlation test, Cohen’s kappa statistics, and ROC curve analysis were conducted. The optimal cutoff points for Minimum Dietary Diversity for Women were determined by selecting the points that maximized the Youden index.

**Results:**

MDD-W had poor positive correlation (*ρ* = 0.19, *p* < 0.001) and poor predictive ability (AUC = 0.62, 95% CI: 0.56, 0.67) (*p* < 0.001) with the Mean Adequacy Ratio in determining micronutrient intake adequacy. The sensitivity and specificity of the MDD-W in the ≥5 food groups standard cutoff were 25.2 and 82.3%, respectively. The optimal cutoff point for MDD-W to predict micronutrient intake adequacy was ≥3 food groups.

**Conclusion:**

Minimum Dietary Diversity for Women had a poor correlation and poor predictive ability in predicting micronutrient intake adequacy. The variations noted in studies and differences from the Food and Agriculture Organization recommendations regarding the cutoff and level of performance of MDD-W in defining micronutrient adequacy warrant further investigation.

## 1 Introduction

Lactating women experience nutritional vulnerability due to the high physiological demands of lactation. Inadequate intake of micronutrients during this phase can have detrimental effects on both the women themselves and the development of their children (1–4). Various global and national efforts have been made to improve the overall quality of women’s diets (5–8). However, a significant proportion of women still experience poor dietary intake with monotonous diets that do not provide sufficient micronutrients, resulting in malnutrition (9–12). To effectively intervene and prioritize actions for Women of Reproductive Age (WRA), it is crucial to enhance the availability and accessibility of high-quality, accurate, and reliable data (13).

Various dietary assessment methods or indicators offer valuable insights into the nutritional adequacy of individuals and populations. However, these approaches can be costly and demand well-trained enumerators and a significant time investment for preparation and data collection. Therefore, they May not be suitable for large-scale surveys. As a result, implementing these methods regularly in Low- and Middle-Income Countries (LMIC) poses significant challenges, hindering the tracking of progress in nutrition interventions (14). To bridge this gap, the Minimum Dietary Diversity for Women (MDD-W) indicator has been developed by the Food and Agriculture Organization of the United Nations (FAO) to evaluate diet quality among WRA. This user-friendly, food-based indicator serves as a tool for monitoring dietary diversity and contributes to the achievement of the Sustainable Development Goals (SDGs) (5, 13, 15).

The MDD-W is a dichotomous indicator of food group diversity that is used as a proxy measure for assessing the adequacy of 11 micronutrients, including vitamin A, B1, B2, B3, B6, B9, B12, C, calcium, iron, and zinc. It determines whether women aged 15 to 49 years have consumed a minimum number of specified food groups within the past 24 h (13). The concept of nutrient adequacy refers to consuming an appropriate amount of vital nutrients necessary to meet the body’s nutritional needs and maintain overall well-being at its best (16). Nutrient adequacy is evaluated through the analysis of quantitative dietary data. It is calculated using the Nutrient Adequacy Ratio (NAR), which compares an individual’s nutrient intake to the nutrient requirement at the Recommended Daily Allowance (RDA) levels to determine their nutrient adequacy (17, 18). Additionally, the Mean Adequacy Ratio (MAR) is used as a comprehensive measure to indicate the overall quality of an individual’s diet (18).

MDD-W has been validated across six countries (Bangladesh, Philippines, Burkina Faso, Mali, Mozambique, and Uganda) to determine the probability of meeting the requirements for 11 essential micronutrients (9, 19). Other studies validated dietary diversity against different numbers of micronutrient intake adequacy among women. The results of studies in Latin America, Thailand, Nigeria, and Burkina Faso exhibited a positive and significant correlation between MDD and the overall MAR of micronutrients, except for the NAR of some nutrients, which displayed a negative correlation. However, the demonstrated conclusion was found to have a low correlation coefficient (20–23).

Moreover, it is crucial to recognize that variations in dietary practices and cultural disparities among different countries worldwide lead to inconsistencies in the findings of studies concerning the actual utilization of MDD-W as proxy indicators to evaluate nutrient intake adequacy (13, 14, 18, 24, 25). The existence of these variations emphasizes the necessity for further validations of MDD-W in the Ethiopian context, where considerable sociocultural differences in dietary consumption practices persist compared to other regions of the world. Bold flavors and distinctive spices, a wide range of dishes, a communal eating approach, and a distinctively dynamic and diversified culinary tradition are all characteristics of Ethiopian food (26, 27). Additionally, there is a lack of evidence regarding the performance of the MDD-W indicator in predicting micronutrient adequacy among Ethiopian lactating women. Therefore, this study aimed to validate the performance of the MDD-W as a predictor of adequate micronutrient intake among lactating women in the North Mecha District, Northwest Ethiopia.

## 2 Materials and methods

### 2.1 Study setting and study design

The study was conducted in the North Mecha District in the Amhara Regional State, Northwest Ethiopia. This particular district is approximately 530 kilometers away from Addis Ababa, the capital city of Ethiopia. Agriculture is prominent in the district, serving as the main means of sustenance for 85% of the local community. The district is widely recognized for its remarkable cultivation of crops such as teff, maize, barley, wheat, beans, and peas. Farmers in the district employ a combination of both rainfall and irrigation techniques to cultivate these crops (28). The district is home to Koga Dam, which can irrigate 7,000 acres of land. The Koga Irrigation and Watershed Management Project, initiated by the government, aims to enhance agricultural productivity and water management in the Koga watershed area of the region. This project focuses on reducing poverty and improving food security. Its execution is anticipated to have a favorable influence on food consumption and dietary practices and also promote socio-economic progress in the region (29). A community-based cross-sectional study design was utilized from February 2nd to 18th, 2023, during the dry season following the autumn harvest and preceding the spring.

### 2.2 Population and eligibility criteria

The source population for this study consisted of lactating women residing in the North Mecha District. On the other hand, the study population consisted of lactating women residing in selected kebeles (local administrative units) within the district. Only lactating women who had lived in the study area for at least six months were eligible to participate. Women who had fasted or were engaged in special events such as festivals or periods of mourning within the last 24 h were also excluded from the study.

### 2.3 Sample size determination and sampling techniques

A sensitivity estimation formula (30) was utilized to determine the sample size based on the following assumptions: a 95% confidence level, a 5% margin of error (d), an anticipated 90% sensitivity (Sn), and a 50% proportion (P). After multiplying in a design effect of 1.5 and accounting for a 10% non-response rate, the final sample size was determined to be 457. The study participants were selected using a multistage sampling technique. Seven kebeles were randomly chosen from a total of 38 kebeles. A systematic random sampling approach was then employed to select the study participants from the chosen Kebeles.


$$N=\frac{{\left(Z_{\alpha /2}\right)}^{2}Sn\;\left(1-Sn\right)}{d^{2}P}$$

### 2.4 Operational and term definitions

#### 2.4.1 Minimum dietary diversity for women

It is a dichotomous indicator that was developed by the FAO to be used as a proxy indicator reflecting the micronutrient adequacy of diets for WRA. It is assessed using ten food groups, which include: grains, white roots and tubers, and plantains; pulses (beans, peas, and lentils); nuts and seeds; milk and milk products; meat, poultry, and fish; eggs; dark green leafy vegetables; other vitamin A-rich fruits and vegetables; other vegetables; and other fruits. If a woman consumed at least five of the ten defined food groups, at least 15 g/day of each food group, within the preceding 24 h, she was considered to have adequate MDD; otherwise, she was considered to have inadequate MDD (13).

#### 2.4.2 Recommended dietary allowances/reference nutrient intake

This represents the recommended daily consumption of nutrients that satisfy the nutritional needs of nearly all (97.5%) lactating women (31).

#### 2.4.3 Nutrient adequacy ratio

It refers to the ratio of a subject’s micronutrient intake to the current RDA for each sex and age category (18).

#### 2.4.4 Mean adequacy ratio

It is a comprehensive measure that serves as an indicator of overall diet quality. It was derived by dividing the sum of all NAR values by the total count of computed micronutrients (18).

#### 2.4.5 Micronutrient intake inadequacy

The occurrence occurred when lactating women consumed less than 100% of the RDA for a specific micronutrient and the NAR for that micronutrient was less than 1 (18).

#### 2.4.6 Overall micronutrient intake inadequacy

The ideal MAR cut-off for nutrient intake inadequacy should be one (100%), which would mean that the intake of all 12 nutrients, namely vitamin A, vitamin B1, vitamin B2, vitamin B3, vitamin B6, vitamin B9, and vitamin B12, vitamin C, calcium, iron, zinc, and selenium, is equal to or greater than the RDA and the requirements for all the nutrients are met. Since no participant had a MAR score of 1 in this study, overall micronutrient intake inadequacy was operationalized to be <0.75 (18, 32–34).

### 2.5 Data collection tools and procedures

A structured interviewer-administered questionnaire was used to collect data on socio-demographic and economic factors and household food security. The data were collected using the Kobo Tool Box, an electronic data collection toolkit. FAO-standardized tools were used to assess the dietary data (14, 35). A team of ten skilled data collectors, supported by two experienced supervisors with expertise in public health nutrition, actively participated in the data collection and supervision process.

### 2.6 24-h dietary recall assessment

Before collecting actual data, home surveillance and market inspections were conducted to gather information about the types of foods consumed, cooking techniques, and kitchen utensils used in the study area. During the surveillance, photographs of household utensils and food portions eaten during a single meal were taken, and a code was assigned to each item. In the nutrition laboratory, utensils used for serving food were standardized using a digital food portion weighing scale and a measuring cylinder with food portions and water (34).

During the data collection process, the respondents were questioned about the utensils they used by referring to a photographic atlas. The atlas contained pictures of various household utensils, such as spoons, ladles, cups, and glasses, as well as food portions. These photographs aided the participants in remembering and determining the types and sizes of the consumed food items. An interactive single multiple-pass 24-h recall method was employed for this purpose. Additionally, the quantities of consumed foods were assessed using household utensils and specific numbers (such as oranges, bananas, mangoes, and potatoes). Foods expressed in numbers were categorized as large, medium, or small (34).

### 2.7 Data quality control

The questionnaire was originally formulated in English and then translated into Amharic before being translated back into English to ensure consistency. The data collection instrument consisted of standard questions developed by the FAO as well as questions adapted from various sources (14, 35). These adapted questions were included in the tool after being validated by experts in the field, and any necessary modifications were made based on their recommendations. A pretest was conducted on approximately 5% of the sample. Supervisors and data collectors received training sessions, and the data collection process was closely monitored to ensure the quality of the data. To assist participants in recalling and identifying the types and quantities of food they had consumed, photographs of food portions and common household utensils such as spoons, ladles, cups, and glasses were utilized.

A multiple-pass, 24-h recall was conducted to collect dietary intake data, consisting of three passes. This procedure involved three sequential stages: first, a “quick list” was created; second, a detailed description of all food and beverage items consumed was recorded; and finally, a review was conducted.

### 2.8 Data processing and analysis

After the data collection process was completed, the food consumption data were converted into nutrient intake data using NutriSurvey 2007 software. The Ethiopian food composition tables were used to evaluate the nutrient values per 100 grams of each food item (36, 37). When certain food items were not specified in the Ethiopian food composition tables, alternative tables from different African countries like Kenya (38) and Tanzania (39) were utilized as points of reference. The analysis was conducted using SPSS version 25, specifically employing NutriSurvey 2007 software to analyze the intake of nutrients.

In 2004, the World Health Organization (WHO) and the FAO established the RDA as a means to compare and evaluate the actual intake of nutrients. The NAR was then calculated to determine the inadequacy of a particular nutrient. Additionally, the MAR was used to assess the overall inadequacy of micronutrient intake. The MAR considered 12 micronutrients that were chosen based on their significance to public health. These micronutrients included vitamin A, thiamin, riboflavin, niacin, vitamin B-6, folate, vitamin B-12, vitamin C, calcium, iron, zinc, and selenium (17, 18).


$$NAR=\frac{\mathrm{Actual intake of the nutrient}\;\mathrm{per}\;\mathrm{day}\;}{RDA\;\mathrm{of that nutrient}}$$

To determine the overall adequacy of micronutrients, the MAR was computed as follows:


$$MAR=\frac{\sum NAR\;\left(\mathrm{each truncated}\;\mathrm{at}\;1\right)}{\mathrm{Number of nutrients}}$$

The NAR was truncated at 1, implying that a nutrient with a superior NAR was unable to compensate for a nutrient with an inferior NAR. To ensure adequacy for NAR, the intake of each nutrient is needed to meet or exceed the RDA. On the other hand, adequacy for the MAR was determined using a ratio. This ratio calculates the sum of the NAR for each nutrient divided by the total number of nutrients, intending to achieve a value of 1 (18, 34). The evaluation of the adequacy of data on micronutrient intake was conducted by employing the Kolmogorov–Smirnov and Shapiro–Wilk tests to assess normality (*p*-value >0.05). The assessment revealed that the data were not distributed normally, and the results were presented using the median and interquartile range.

The validity of MDD-W was assessed by comparing it to MAR, which serves as a gold standard for high accuracy. Since the MAR distribution was skewed, Spearman’s rank correlation test was employed to investigate the correlation between MDD-W and MAR. The strength of the correlation was interpreted using the correlation coefficient as follows: poor (0–0.29), fair (0.30–0.59), moderate (0.60–0.79), and strong (0.80–1.00) correlation (40, 41). To assess the level of agreement between MDD-W and MAR, Cohen’s kappa statistics were employed. The kappa scores were interpreted as follows: poor agreement (<0.00), slight agreement (0.00–0.20), fair agreement (0.21–0.40), moderate agreement (0.41–0.60), substantial agreement (0.61–0.80), and almost perfect agreement (0.81–1.00) (42).

Additionally, sensitivity and specificity analyses were carried out to assess the accuracy of MDD-W in correctly identifying lactating women with high MAR values. The Area Under the Curve (AUC) was calculated using the Receiver Operating Characteristic (ROC) curve based on the nutrient adequacy as either yes (MAR ≥0.75) or no (MAR <0.75). Additionally, further analyses were conducted to assess the performance of the MDD-W at different MAR thresholds, which were set between 0.50 and 0.85. The AUC values were then interpreted according to predefined criteria, which categorized them as fail (0.5–0.6), poor (0.6–0.7), fair (0.7–0.8), good (0.8–0.9), or excellent accuracy (0.9–1.0) (43). The optimal cutoff points were identified by selecting the points that maximized the Youden J statistic (sensitivity + specificity – 1) (the larger the better) (44). A *p*-value less than 0.05 was considered statistically significant. Texts, tables, and graphs were used to present the final results.

## 3 Results

### 3.1 Socio-demographic and socioeconomic characteristics

A total of 430 lactating women participated in the study, with a 94.1% response rate and a mean age of 29.46 ± 5.55 years. Three hundred nineteen (74.2%) participants were unable to read and write in terms of their educational status. The mean family size was 5.81 ± 1.90. All study participants were followers of the Orthodox Christian religion (Table 1).

**Table 1:** Socio-demographic characteristics among lactating women in North Mecha District, Northwest Ethiopia, 2023 (N = 430).

Variables	Frequency	Percentage
Maternal age in years		
18–25	119	27.7
26–35	258	60.0
36–50	53	12.3
Maternal education		
Unable to read and write	319	74.2
Primary school incomplete	40	9.3
Primary school completed	42	9.8
Secondary school completed	24	5.6
University or college completed	5	1.2
Maternal occupation		
Farmer	333	77.4
Merchant	17	4.0
Housewife	78	18.1
Employee	2	0.5
Maternal marital status		
Married	423	98.4
Widowed	7	1.6
Partner education (423)		
Unable to read and write	193	44.9
Primary school incomplete	165	38.4
Primary school completed	44	10.2
Secondary school completed	17	4.0
University or college completed	4	0.9
Partner occupation (423)		
Farmer	390	90.7
Merchant	15	3.5
Student	4	0.9
Daily laborer	7	1.6
Other^#^	7	1.6
Family size		
≤4	123	28.6
5–7	224	52.1
≥8	83	19.3
Parity		
≤2	123	28.6
3–5	209	48.6
≥6	98	22.8

^#Driver, soldier, veterinarian.^

### 3.2 Minimum dietary diversity and nutrient adequacy of lactating women

**Figure 1:**
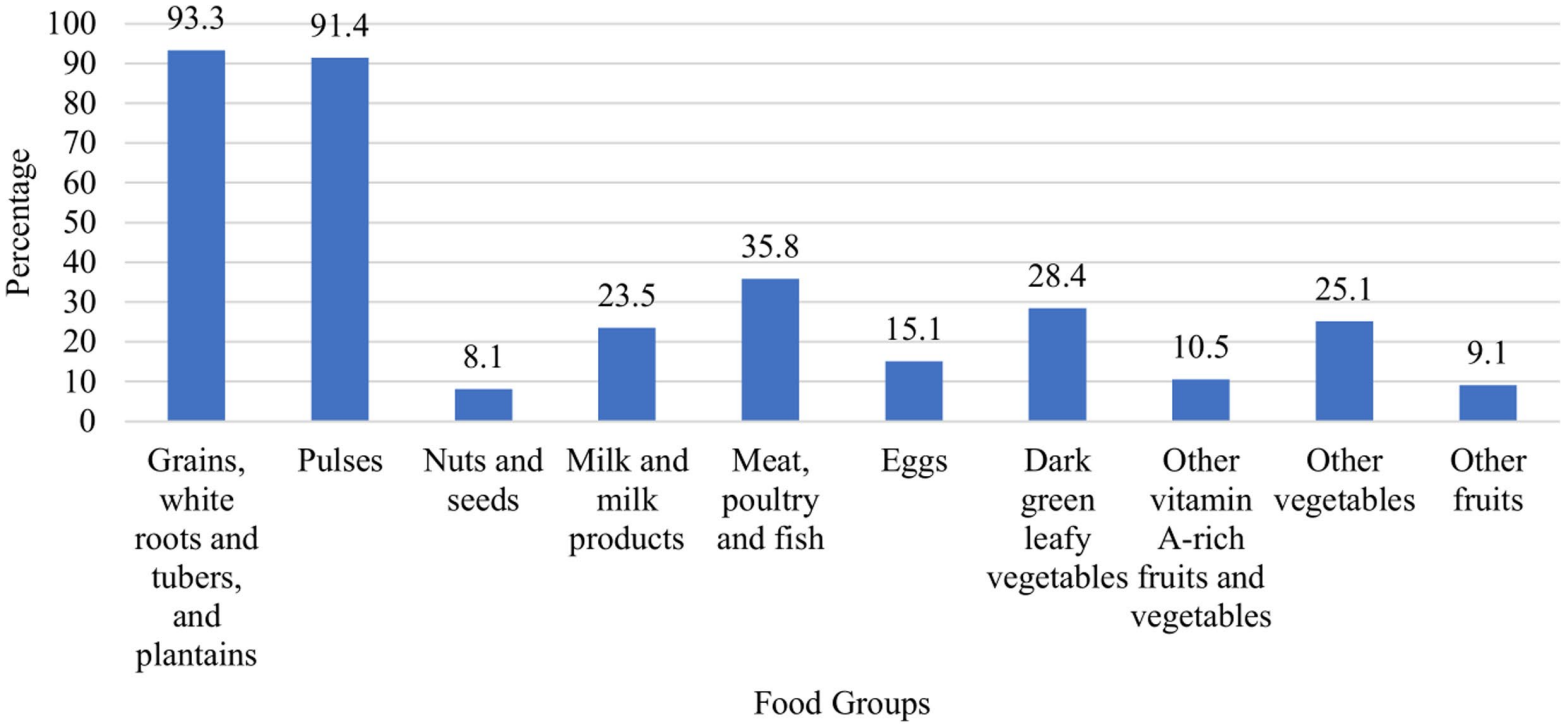
Number of consumed food groups among lactating women in North Mecha District, Northwest Ethiopia (*N* = 430).

**Figure 2:**
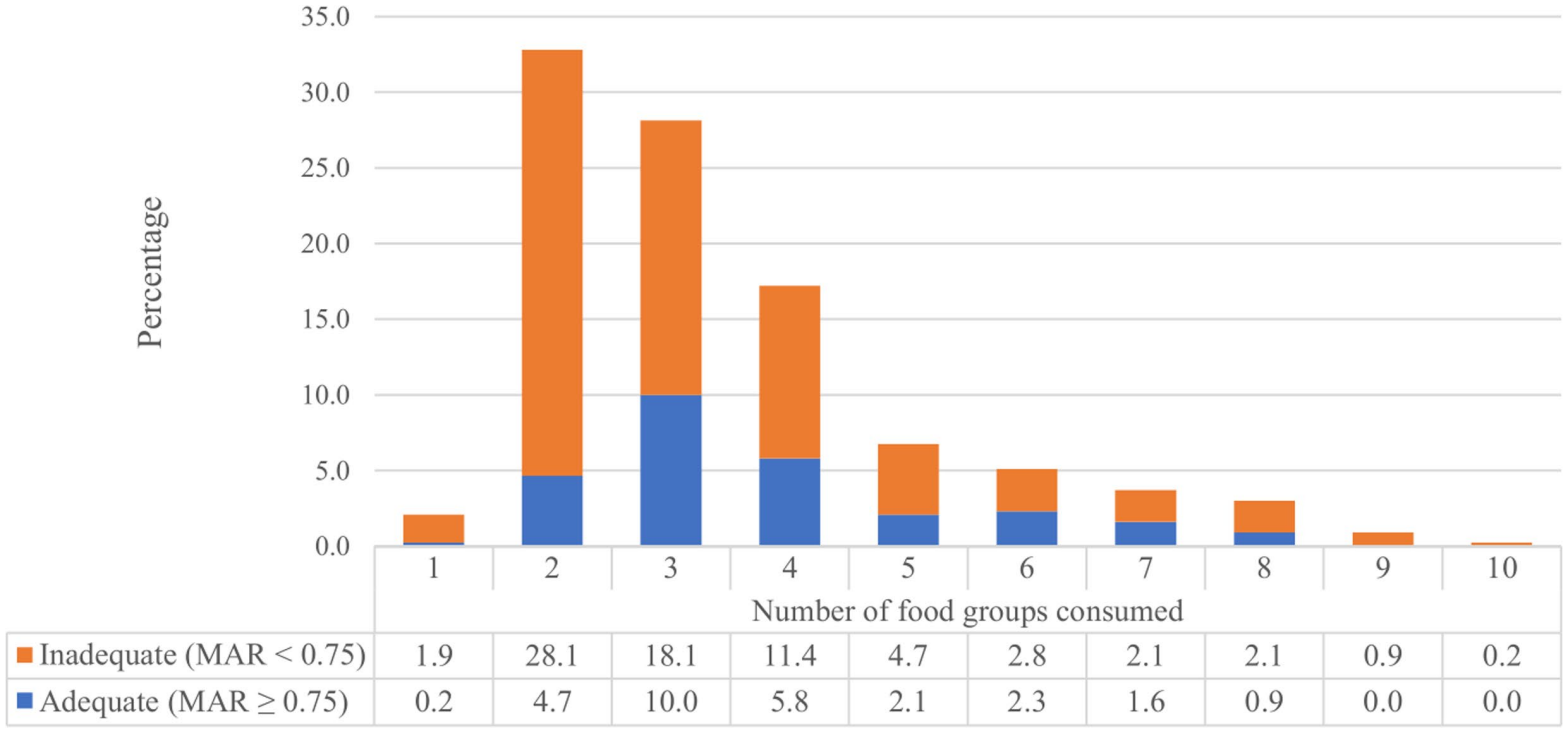
Types of consumed food groups among lactating women in North Mecha District, Northwest Ethiopia (*N* = 430).

The range of food groups consumed varies from a minimum of one food group to a maximum of ten food groups. The prevalence of the MDD-W score (≥5 food groups) was 19.8% (95% CI: 16.1, 23.9). The median MDD-W score was 3.0 ± 2.0 (Figure 1). A large majority of study participants had consumed starchy staple foods and pulses (beans and peas), while the least consumed food group was nuts and seeds (Figure 2). The overall prevalence of adequate micronutrient intake, defined as MAR ≥0.75, was 27.7% (95% CI: 23.5, 32.2). This prevalence was calculated among the 11 nutrients after excluding iron, which was adequate for all participants.

### 3.3 Validation of the MDD-W in predicting adequacy of micronutrient intake

**Figure 3:**
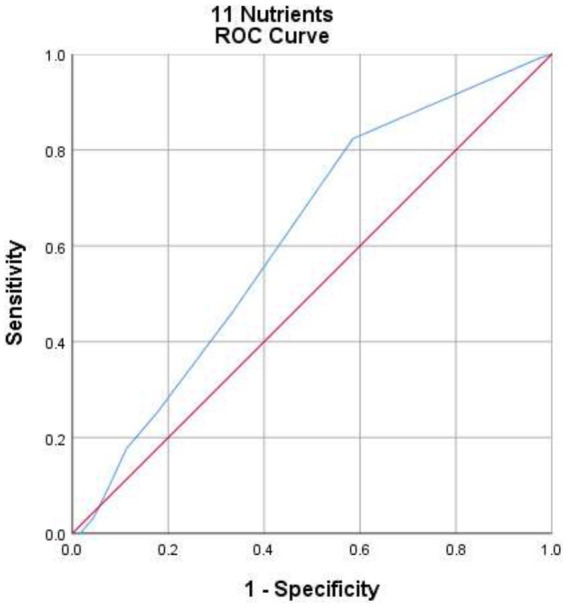
The ROC curve illustrates the performance of dietary diversity for women in predicting adequate micronutrient intake among lactating women in North Mecha District, Northwest Ethiopia, 2023 (MAR ≥ 0.75) (*N* = 430).

The Spearman correlation test revealed a statistically significant poor positive correlation between the MDD-W and the MAR in determining the adequacy of micronutrient intake (*ρ* = 0.19, *p* < 0.001). According to the ROC analyses, MDD-W exhibited poor predictive ability for micronutrient adequacy concerning 11 micronutrients at MAR ≥0.75 (AUC = 0.62, 95% CI: 0.56, 0.67) (*p* < 0.001) (Figure 3; Table 2).

**Table 2:** Summary of dietary diversity for women indicator characteristics at various MAR thresholds among lactating women in North Mecha District, Northwest Ethiopia, 2023 (*N* = 430).

Threshold	*p*	AUC	MDD optimal cutoff (≥)	Value at the optimal cutoff			
				Sensitivity (%)	Specificity (%)	J	Kappa
MAR ≥ 0.50	0.11*	0.60 (0.51, 0.70)*	3	66.6	0.50	0.17	0.071*
MAR ≥ 0.65	0.11*	0.56 (0.51, 0.62)*	3	69.9	41.4	0.11	0.116*
MAR ≥ 0.67	0.14**	0.58 (0.52, 0.63)**	3	72.4	43.1	0.15	0.157**
MAR ≥ 0.75	0.19***	0.62 (0.56, 0.67)***	3	82.4	41.5	0.24	0.168***
MAR ≥ 0.85	0.12*	0.63 (0.54, 0.72)*	3	91.7	36.5	0.28	0.047**

*ρ*: spearman rank correlation coefficient rho, AUC: area under the curve, J: youden index, MDD: minimum dietary diversity, MAR:mean adequacy ratio. *p value < 0.05, **p value < 0.01, ***p value < 0.001.

### 3.4 Determination of the cutoff for dietary diversity

The optimal cutoff was determined to be food groups of three, and this remained consistent regardless of the MAR threshold set between 0.50 and 0.85. The agreement levels were generally slight for each MAR threshold. However, the highest level of agreement was achieved when the MAR value was ≥0.75 (Table 2).

The MDD-W score (≥5 food groups), which is a proxy indicator developed by FAO to reflect micronutrient adequacy, showed a sensitivity of 25.2% and a specificity of 82.3%. The optimal cutoff point for MDD-W to predict micronutrient intake adequacy based on the findings of the present study, determined by considering the Youden index, was ≥3 food groups, resulting in a sensitivity of 82.4% and a specificity of 41.5%. The Kappa statistics also demonstrated a relatively higher Kappa value when examining the dietary diversity of ≥3 food groups. This suggests a slight agreement between the consumption of ≥3 food groups and meeting the essential micronutrient requirements (Table 3).

**Table 3:** Summary of dietary diversity for women indicator characteristics for 10 food groups among lactating women in North Mecha District, Northwest Ethiopia, 2023 (*N* = 430).

Food groups	Sensitivity (%)	Specificity (%)	PPV (%)	NPV (%)	J	Kappa
≥1	100	0	27.7	0	0	0.000
≥2	99.2	2.6	28.0	88.9	0.02	0.010
≥3	82.4	41.5	35.0	86.0	0.24	0.168***
≥4	46.2	66.6	34.6	76.4	0.13	0.116*
≥5	25.2	82.3	35.3	74.2	0.08	0.083
≥6	17.6	88.7	37.5	73.8	0.06	0.076
≥7	9.2	92.6	32.4	72.7	0.02	0.024
≥8	3.4	95.5	22.2	72.1	−0.01	−0.015
≥9	0	98.4	0.0	72.0	−0.02	−0.023
10	0	99.7	0.0	72.3	<−0.01	−0.005

PPV: predictive value positive, NPV: negative predictive value, J: youden index. *p value < 0.05, **p value < 0.01, ***p value < 0.001.

Compared to all the studied micronutrients, dietary diversity for women demonstrated relatively high performance in predicting nutrient intake adequacy for Vitamin B12 (NAR = 1). The Spearman rank correlation indicated that dietary diversity for women showed a fair positive correlation with vitamin B12 intake adequacy and a poor positive correlation with the adequacy of other micronutrients that had a significant correlation (vitamin B2, B3, B12, zinc, and selenium). Except for vitamin B1, which showed a poor negative correlation, the AUC of all micronutrients with significant values ranged from 0.57 to 0.69, with the highest score exhibited for vitamin B12. Taking into consideration the Youden index, the optimal cutoff point for MDD-W to predict micronutrient intake adequacy was determined to be ≥3 food groups for all micronutrients with significant values, except vitamin B1 (Table 4).

**Table 4:** Summary of dietary diversity for women indicator characteristics for each micronutrient among lactating women in North Mecha District, Northwest Ethiopia, 2023 (NAR =1) (*N* = 430).

Micronutrient	*ρ*	AUC	MDD optimal cutoff (≥)	Value at the optimal cutoff			
				Sensitivity (%)	Specificity (%)	Youden Index	Kappa
Vitamin A	0.09	0.59 (0.51,0.69)*	3	77.2	36.7	0.14	0.052*
Vitamin B1	−0.19***	0.38 (0.32,0.44)***	2	98.6	3.4	0.02	0.027
Vitamin B2	0.16**	0.61 (0.55,0.66)***	3	76.0	42.9	0.19	0.177***
Vitamin B3	0.19***	0.64 (0.58,0.70)***	3	86.9	40.2	0.27	0.144***
Vitamin B6	0.06	0.55 (0.48,0.62)	5	21.3	87.2	0.09	0.036
Vitamin B9	0.08	0.57 (0.52,0.63)*	3	72.8	39.8	0.13	0.113**
Vitamin B12	0.33***	0.69 (0.63,0.75)***	3	93.4	41.0	0.34	0.167***
Vitamin C	0.05	0.59 (0.43,0.76)	3	85.7	35.2	0.21	0.010
Calcium	0.06	0.54 (0.44,0.64)	3	71.0	35.3	0.06	0.013
Iron	–	–	–	–	–	–	–
Zinc	0.13**	0.58(0.52,0.64)**	3	69.0	44.4	0.13	0.125**
Selenium	0.15**	0.62(0.55,0.70)**	3	67.9	52.5	0.21	0.124**
11 Nutrients	0.19***	0.62(0.56, 0.67)***	3	82.4	41.5	0.24	0.168***

*ρ*: spearman rank correlation coefficient rho, AUC: area under the curve, J: youden index. *p value < 0.05, **p value < 0.01, ***p value < 0.001.

## 4 Discussion

This study aimed to validate the performance of the MDD-W as a predictor of adequate micronutrient intake among lactating women in the North Mecha District, Northwest Ethiopia. The present study revealed that the occurrence of a satisfactory MDD-W (≥5 food groups) as suggested by FAO was 19.8% within the 24 h preceding the survey. The findings of this study demonstrate a lower rate when compared to the most recent findings from studies conducted in Ethiopia (45–50). The potential difference could be explained by various factors, including seasonal variations that affect food availability, particularly vegetables, differences in study settings such as urban versus rural areas, divergent sociocultural practices in dietary consumption, and disparities in nutrition-related knowledge and attitudes.

The MAR for lactating women fell within the range of 0.29 to 0.94, indicating that none of the participants reached the RDA for all essential nutrients and had a MAR of one, which is supported by other studies (51, 52). This study also showed that the overall prevalence of micronutrient intake adequacy (MAR ≥0.75) among lactating women was 27.7%. This is significantly lower than the findings of the study conducted in Bahir Dar City, Ethiopia (52), as well as in rural areas of Indonesia (53). The potential explanation for the low adequacy of micronutrient intake includes factors such as the dominant consumption of cereals with low micronutrient density, such as teff, maize, and sorghum, and seasonal variations. Moreover, there is insufficient consumption of food items from other food groups, such as animal-source foods, pulses, fruits, vegetables, and nuts and seeds, which are rich in essential micronutrients (54). In contrast to other studies, the variation observed in this study can also be attributed to disparities in study settings, encompassing rural and urban areas (52), as well as disparities in dietary reference intakes, such as RDA and Estimated Average Requirement (EAR) (31). This discrepancy can be observed as one instance of disparity between studies employing RDA (21, 52, 55) and EAR (51, 53, 56–59).

A variety of validation tests were utilized to assess the validity of the MDD-W. The Spearman correlation analysis of this study indicated a statistically significant but poor positive correlation between MDD-W and the adequacy of intake for 11 micronutrients (MAR ≥0.75). Additionally, in the ROC analyses, MDD-W exhibited poor predictive ability for micronutrient adequacy. Likewise, a Latin American study revealed that the Dietary Diversity Score exhibited a significant weak positive correlation with the MAR score (20). Additionally, this study finding was relatively comparable to a study conducted in Burkina Faso, which found a moderate correlation between MDD-W and the adequacy of micronutrient intake in lactating women (23). Other studies conducted in Nigeria and Thailand also revealed a significant correlation between the dietary diversity score and nutrient adequacy. It was observed that as the dietary diversity score increased, so did the nutrient intake adequacy (21, 22). Furthermore, studies conducted among WRA from low- and middle-income countries have consistently revealed a notable correlation between MDD-W and Mean Probability of Adequacy (MPA) at each respective site (9, 19).

The MDD-W score (≥5 food groups), a proxy indicator endorsed by the FAO to assess the adequacy of micronutrients (13), exhibited a sensitivity of 25.2% and a specificity of 82.3%, according to the current study. This suggests that MDD-W is a specific measure for assessing micronutrient adequacy at the defined threshold. It indicates that when the MDD-W indicator is used with a cutoff of ≥5 food groups, it is more likely to ensure that those who have adequate MDD-W and test positive are most likely to have adequate intake of micronutrients according to the MAR score. As a result, it reduces the likelihood of false positive results, such as incorrectly identifying women as having micronutrient adequacy when they have a low probability of it, especially if their diet consists of a less diverse range of foods from at most four different food groups. On the other hand, the low sensitivity of the indicator suggests that it performs poorly in identifying women with adequate levels of micronutrient adequacy and has a high chance of producing false negative results, failing to identify those who have adequate micronutrient intake at the defined MDD-W cutoff.

In comparison to the findings of the Women’s Dietary Diversity Project (WDDP II), led by the FAO, the sensitivity of the MDD-W indicator (≥5 food groups) was significantly lower in the present study than the sensitivity of a similar indicator calculated from 10 food groups (restricted) with an MPA >0.7, compared to the results of each study site in the included countries. On the contrary, the specificity of the MDD-W indicator (≥5 food groups) in the present study was higher than the specificity results obtained for the similar indicator calculated from 10 food groups (restricted) with an MPA >0.7 among women, compared by study site in each country (19).

The optimal cutoff point for MDD-W to predict micronutrient intake adequacy based on the findings of the present study, determined by considering the Youden index, was ≥3 food groups, resulting in a sensitivity of 82.4% and a specificity of 41.5%. The Kappa statistics also demonstrated a relatively higher Kappa value when examining the dietary diversity of ≥3 food groups. The finding suggests a slight agreement between the consumption of ≥3 food groups and meeting the essential micronutrient requirements. Similarly, the optimal cutoff point for MDD-W in predicting the adequacy of micronutrient intake was found to be ≥3 food groups for all micronutrients with significant values (vitamin A, B2, B3, B12, zinc, and selenium), except for vitamin B1. In line with the slight agreement observed between the MDD-W (≥3 food groups) and the overall adequacy of micronutrients using MAR, the MDD-W (≥3 food groups) for individual micronutrients also showed slight agreement with the adequacy of each micronutrient using NAR. The best cutoff determination, considering the maximum Youden index value, revealed a better sensitivity for the MDD-W (≥3 food groups) indicator than for the MDD-W (≥5 food groups) indicator, which had high specificity but low sensitivity.

The cutoff derived from this study is lower than what has been revealed by other studies. A study in Burkina Faso demonstrated the effectiveness of MDD-W in accurately predicting the MPA value >0.6 in women who were not pregnant or breastfeeding. Moreover, it was observed that setting a threshold of a four-food-group cutoff for MDD-W for a food group score of 10 resulted in a better balance between sensitivity, specificity, and the accuracy of classification compared to MDD-W with a five-food-group cutoff, thereby reflecting improved adequacy of micronutrients. However, this study highlights that the levels of MPA for pregnant and breastfeeding women were insufficient to determine the best cutoff points effectively (23). Another study conducted among WRA from low- and middle-income countries demonstrated that using a cutoff of ≥5 food groups in the measurement of MDD-W yielded the best balance between sensitivity and specificity across study sites. This was observed for both indicators derived from nine and ten food groups, with a MPA >0.6 (9).

One possible explanation for this variation could be related to the threshold of MAR selected to define the level of adequacy required to be considered satisfactory. When validating the MDD-W indicator with a uniform cutoff, sensitivity increases as the MAR cutoff increases, while specificity decreases. This had little effect on determining the best balance of sensitivity and specificity and defining the best cutoff. Additionally, cultural disparities, methodological discrepancies, individual dietary patterns, and socioeconomic influences all play a significant role in shaping these variations.

However, since lactating women have higher micronutrient requirements than other WRA, a higher cutoff of MDD is expected to predict MAR ≥0.75 compared to other study findings that evaluated MDD at MPA >0.6 and concluded the best cutoff of four or five food groups (9, 19, 23), including what is endorsed by FAO (13). Furthermore, when taking into account the necessary level of micronutrient requirements to meet the RDA as utilized in this particular nutrient analysis study, it is worth noting that the requirement level exceeds that of micronutrients necessary to meet the EAR as employed in other research studies (9, 19, 23). This elevated requirement level consequently leads to a higher MDD-W cutoff to achieve RDA-based adequacy. Conversely, it is significant that this anticipation contrasts with the findings of previous studies once again.

The variations noted in studies and differences from FAO recommendations regarding the cutoff and level of performance of MDD-W in defining micronutrient adequacy have several implications. These include improved identification of individuals with adequate micronutrient intake, effective allocation of resources, enhanced monitoring and evaluation, and policy considerations. Therefore, as countries are implementing the FAO-endorsed MDD-W guideline in their health systems, discrepancies in its performance have been observed in various studies, including the current one. Hence, further investigation is required in different settings with diverse dietary consumption practices to fully comprehend the reasons behind these controversies and adapt management strategies accordingly. Along with the relevant findings of this study in the northwest part of Ethiopia, additional comprehensive analysis of dietary intake data is necessary for other parts of Ethiopia, a country with a diverse population and varying dietary consumption practices.

This community-based study validated the performance of MDD-W, which is endorsed by FAO for low- and middle-income countries (13), in defining micronutrient intake adequacy. The study specifically focuses on lactating women in Ethiopia, as their consumption plays a significant role in determining the nutritional status of both the mother and child. Also, the study addresses a lack of evidence regarding the performance of the MDD-W indicator in Ethiopia, where considerable sociocultural differences in dietary consumption practices persist compared to other regions of the world. Nevertheless, relying solely on a single 24-h recall May introduce potential inaccuracies due to variations in daily dietary patterns and memory biases. To minimize these biases, significant efforts have been made to incorporate standardized quality-control procedures throughout the entire study process. Well-trained nutrition experts have been involved to ensure accuracy and consistency. Additionally, days of special events are excluded from the data collection process to ensure that the findings reflect regular dietary patterns.

## 5 Conclusion

A statistically significant, yet poor, positive correlation was found between MDD-W and MAR when determining the adequacy of micronutrients. Moreover, in the ROC analyses, MDD-W exhibited poor predictive ability for micronutrient adequacy. The optimal cutoff point for MDD-W in predicting adequate intake of micronutrients was established as ≥3 food groups. The variations noted in studies and differences from the FAO recommendations regarding the cutoff and level of performance of MDD-W in defining micronutrient adequacy are crucial and have several implications. These include enhanced monitoring and evaluation, effective allocation of resources, and policy considerations. In light of this consideration, along with the pertinent findings of this study in the northwest part of Ethiopia, further investigations are needed to determine the reasons behind the deviations and reexamine the recommendation of using MDD-W as a proxy indicator for predicting micronutrient adequacy. This should incorporate more study sites in a multicounty context, including Ethiopia, a country with a diverse population and varying dietary consumption practices.

## Data Availability

The raw data supporting the conclusions of this article will be made available by the authors, without undue reservation.
